# Supplementary material for: MIDASim: a fast and simple simulator for realistic microbiome data
Source: Microbiome. 2024 Jul 22;12:135. doi: 10.1186/s40168-024-01822-z (PMC11264979; doi:10.1186/s40168-024-01822-z)
Supplement: Supplementary file 2 — Supplementary Material 1. [file 40168_2024_1822_MOESM1_ESM.pdf]

Supplementary File: Statistical Analyses

We compared the simulated data from each method to the template data using several measures. First, we concatenated the template data with a simulated dataset from each method, and defined a binary variable to differentiate the template and simulated data. We tested the significance of this variable using PERMANOVA [24], which tests for shifts in the between-observation distances. Our PERMANOVA tests used the Jaccard distance as well as the Bray-Curtis distance, which are both commonly used in microbiome data analyses. The Jaccard distance uses only presence-absence information in the data, and thus can assess how similar $\widetilde{\mathbf{Z}}$ and $\mathbf{Z}$ are, while the Bray-Curtis distance accounts for both the presence-absence and relative abundance information and can be used to assess the simulation of $\widetilde{\pi}$. We also compared the alpha diversity of the simulated data and template data. The simulated communities were compared to the template in terms of observed richness and Shannon Index, and the differences in diversity were tested by Kruskal-Wallis tests. The observed richness is simply the number of observed taxa, while Shannon Index additionally considers evenness-the relative abundances of taxa-when quantifying diversity. To suppress random variability, we repeated the comparison of alpha-diversity and beta-diversity using 20 simulated datasets from each of the four methods. Finally, we compared the methods visually, using ordination and PCoA, as well as boxplots of alpha diversity values, using a single simulated data set for each method.

We next compared the simulation approaches in terms of their β-dispersion, by comparing whether the distribution of distances from each observation to the sample centroid was the same in the simulated and template data. We calculated distances to the centroids using the betadisper

function in R package `vegan` [37]. We used the Kolmogorov-Smirnov (K-S) test to compare these empirical distributions. We again averaged results over 20 simulation replicates to suppress random variability. We also compared the alpha diversity of the template and simulated data, as measured by the species richness (number of observed taxa) and the Shannon entropy.

Finally, we evaluated the performance of our approach to generating data with different library sizes by rarefying our template datasets, then using the approach described in section 2.3 to increase the library size to that of the original template data. Thus, we can compare the resulting simulated data to the original template data. Specifically, for each template, the observed counts for each subject were rarefied (subsampled without replacement) to remove 10% of the observed counts. The rarefied data are then treated as the template data in MIDASim, and the target library size is the original library size.

Supplementary File: Tables and Figures

Table S1: Average p-values for comparing alpha and beta diversities in MIDASim simulated data (20 replicates) versus template data, without removal of rare taxa.

		**Beta-Diversity***		**Alpha-Diversity****			
Data	Method	Jaccard	Bray-Curtis	Richness t	Richness KS	Shannon t	Shannon KS
IBD	MIDASim (nonparametric)	0.9938	1.0000	0.6536	0.2756	0.5472	0.6339
	MIDASim (parametric)	0.5511	0.9813	0.6388	0.2460	0.0946	0.0459
MOMS-PI	MIDASim (nonparametric)	0.1367	0.9099	0.6152	0.0012	< 0.0001	< 0.0001
	MIDASim (parametric)	0.0017	0.0010	0.2830	0.1799	< 0.0001	< 0.0001

* Beta-diversity comparisons were conducted using PERMANOVA.

** Alpha-diversity comparisons were conducted using both t-test and the Kolmogorov-Smirnov (KS) test.

Table S2: Summary statistics of the IBD and MOMS-PI datasets used in comparison after filtering.

**Dataset**	**Sample size**	**# of taxa**	**Log10 Library size mean (min, max)**	**% of zeros**	**CV* mean (min, max)**
IBD	146	614	4.22 (3.51, 4.50)	85.09	6.24 (0.90, 11.98)
MOMS-PI	517	1146	4.61 (3.50, 5.78)	95.25	13.58 (1.65, 22.72)

* CV is the coefficient of variation of observed OTU counts for each taxon.

Table S3: Summary of CPU time and memory usage for fitting templates and simulating one dataset with varying taxa (J) and sample size (n). Template sizes range from 100 to 1000 taxa, and sample sizes vary between 100 and 5000. Simulated datasets match the size of the corresponding templates in each J and n combination.

Mode	Sample size	Time s			Memory allocation (MB)		
		$J = 100$	$J = 500$	$J = 1000$	$J = 100$	$J = 500$	$J = 1000$
nonparametric	$n = 100$	3.3	18.8	57.8	182.4	1261.2	3212.0
	$n = 1000$	16.4	105.7	337.5	1529.2	6781.2	16574.8
	$n = 5000$	73.3	517.6	1606.0	8138.9	40427.4	82572.2
parametric	$n = 100$	4.3	25.2	70.1	190.1	1298.4	3262.3
	$n = 1000$	15.4	111.4	338.8	1509.7	8220.8	17454.1
	$n = 5000$	71.4	526.0	1569.5	7768.5	39969.2	81411.3

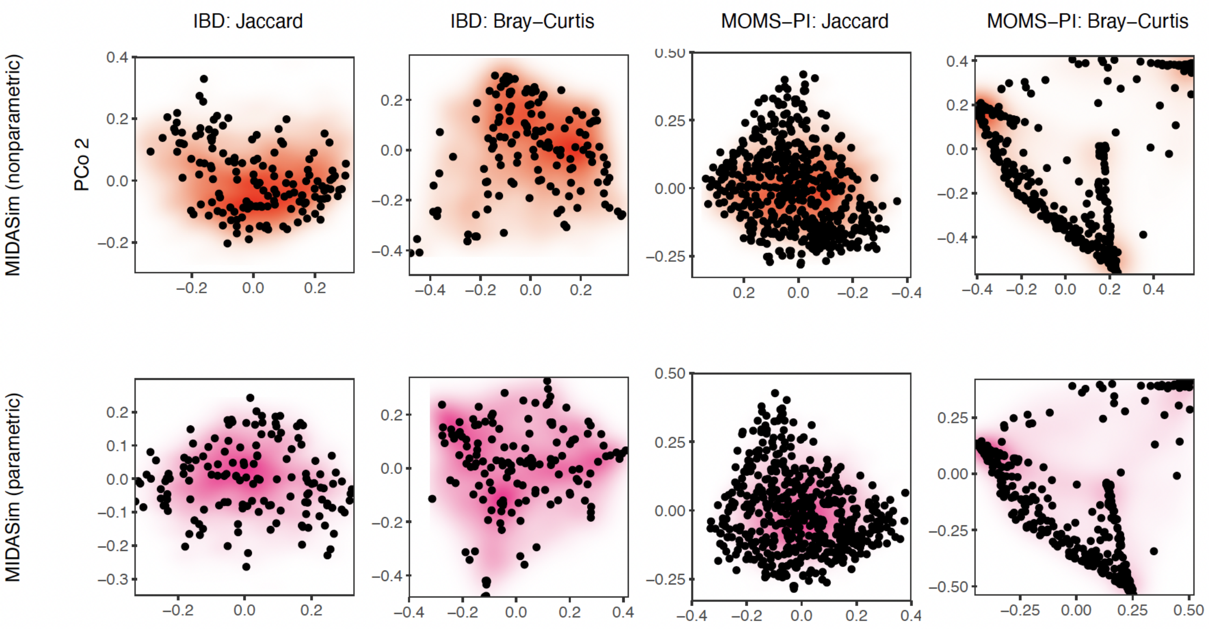

Figure S1: Principal Coordinates plots (PCoA) of the simulated and original microbiome community. The colored density map is plotted based on 20 replicates of simulated communities by MIDASim, with darker coloring associated with higher density of simulated values. Black points represent the original community.

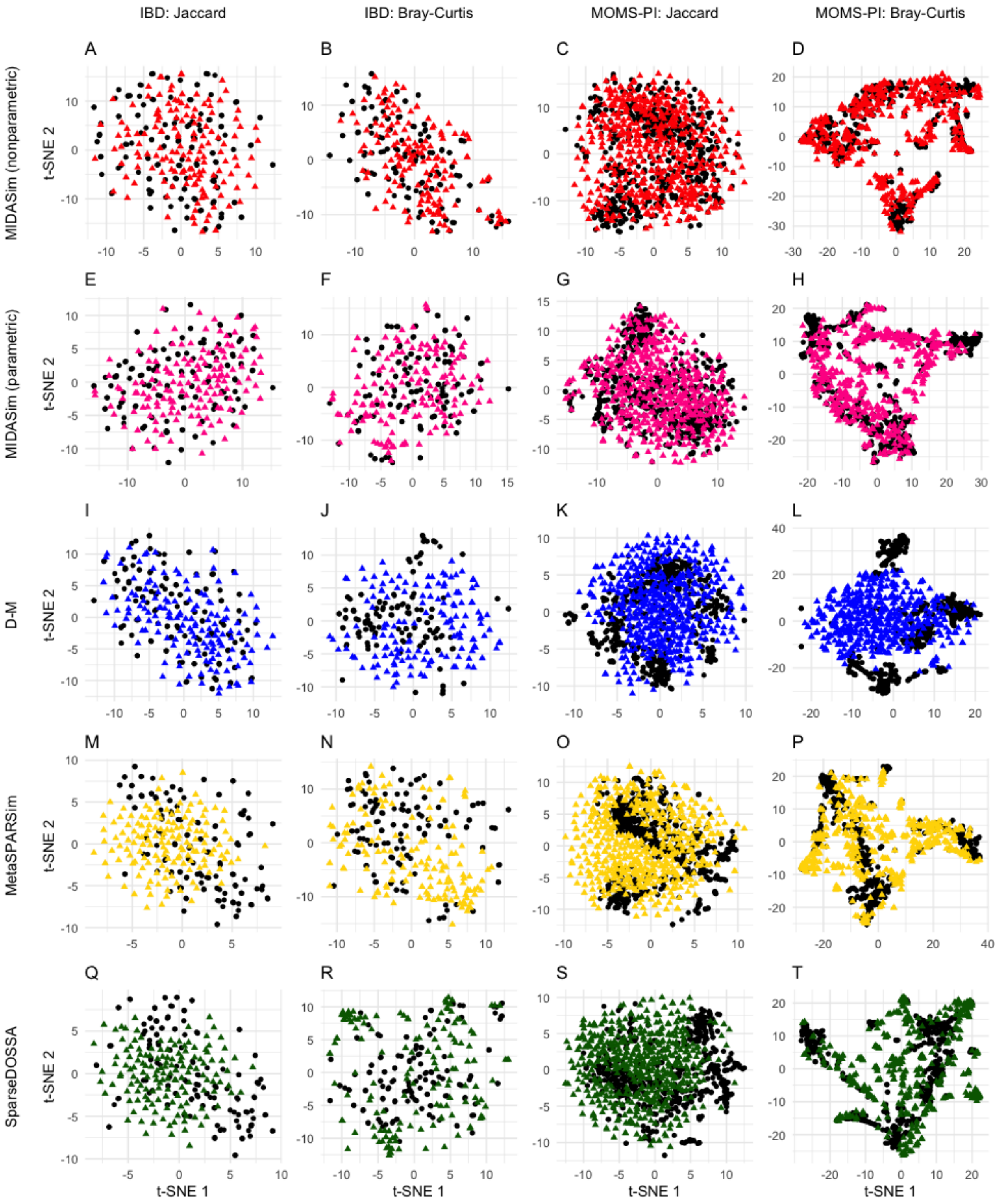

Figure S2: Plots of t-distributed stochastic neighbor embedding (t-SNE) of the simulated and original community. Each row corresponds to one method. The left two columns are the plots for the IBD data, and the right two columns are the plots for the MOMS-PI data. Black points: samples from original data. Colored points: samples from the simulated data with red being MIDASim with nonparametric model, pink being MIDASim with parametric model, blue being D-M, yellow being MetaSPARSim, and green being SparseDOSSA.

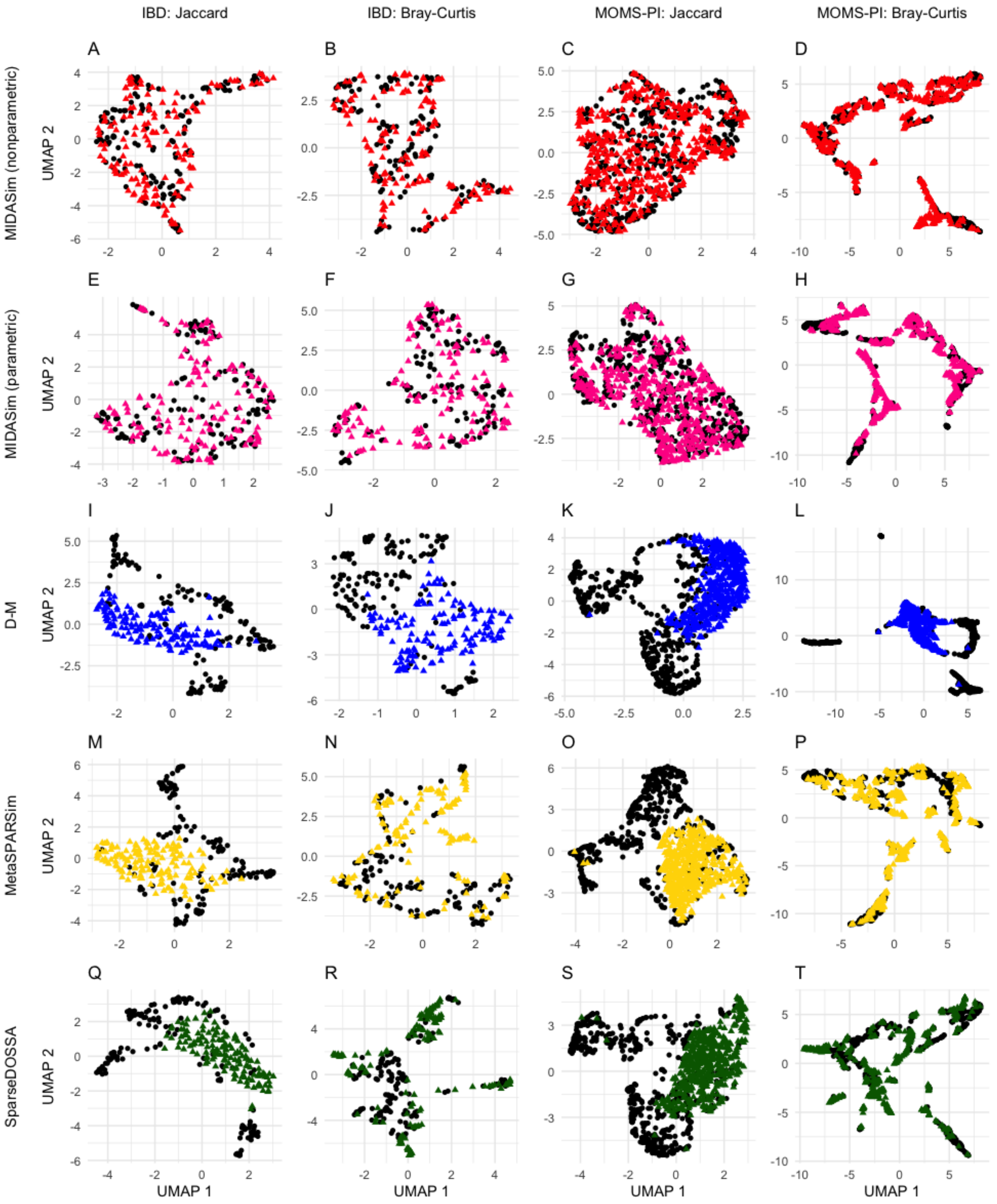

Figure S3: Plots of Uniform Manifold Approximation and Projection (UMAP) of the simulated and original community. Each row corresponds to one method. The left two columns are the plots for the IBD data, and the right two columns are the plots for the MOMS-PI data. Black points: samples from original data. Colored points: samples from the simulated data with red being MIDASim with nonparametric model, pink being MIDASim with parametric model, blue being D-M, yellow being MetaSPARSim, and green being SparseDOSSA.

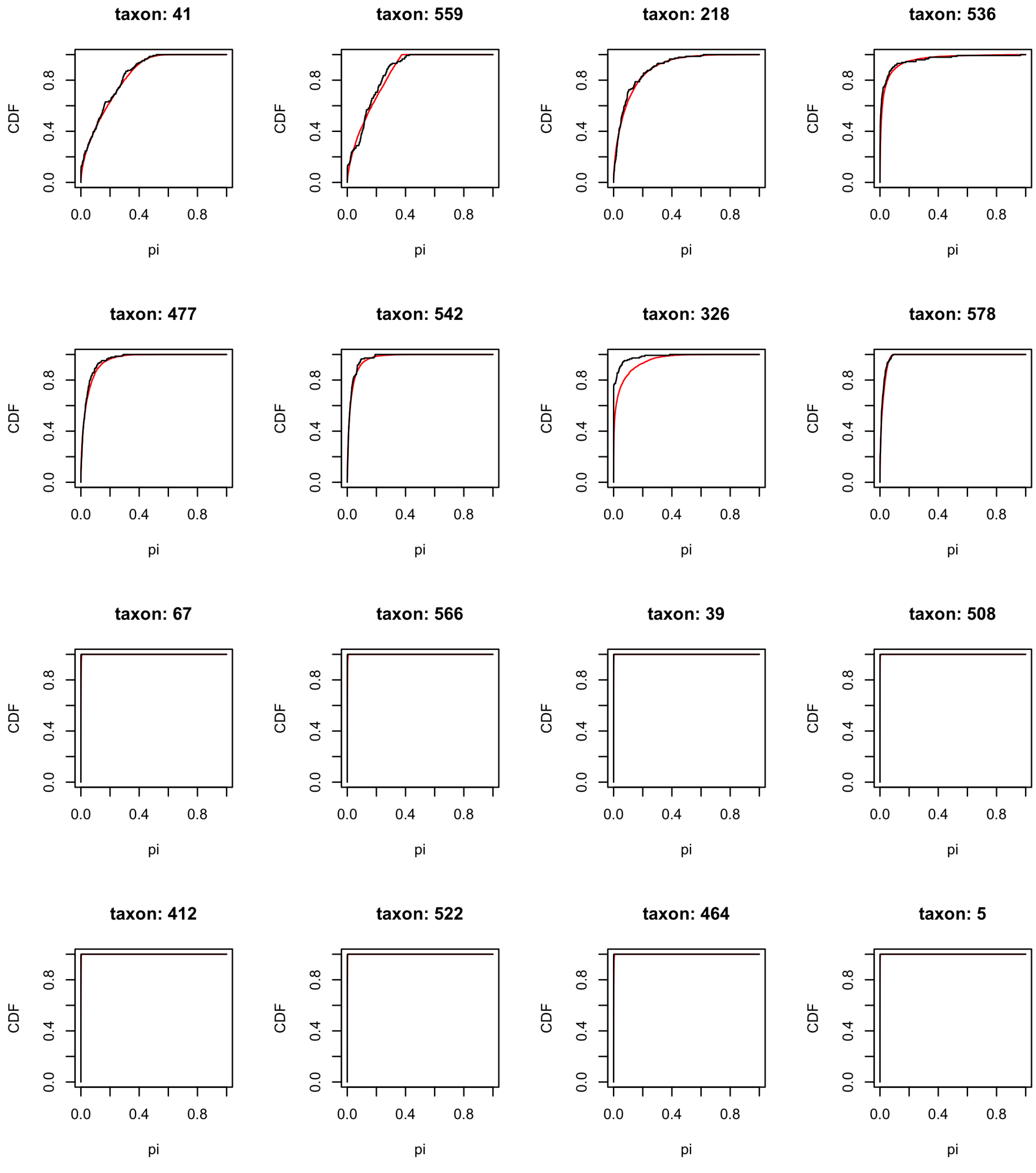

Figure S4: Comparison of the predicted (red) and empirical (black) estimates of the CDF of relative abundance for the top 8 and moderately abundant 8 taxa in IBD dataset.

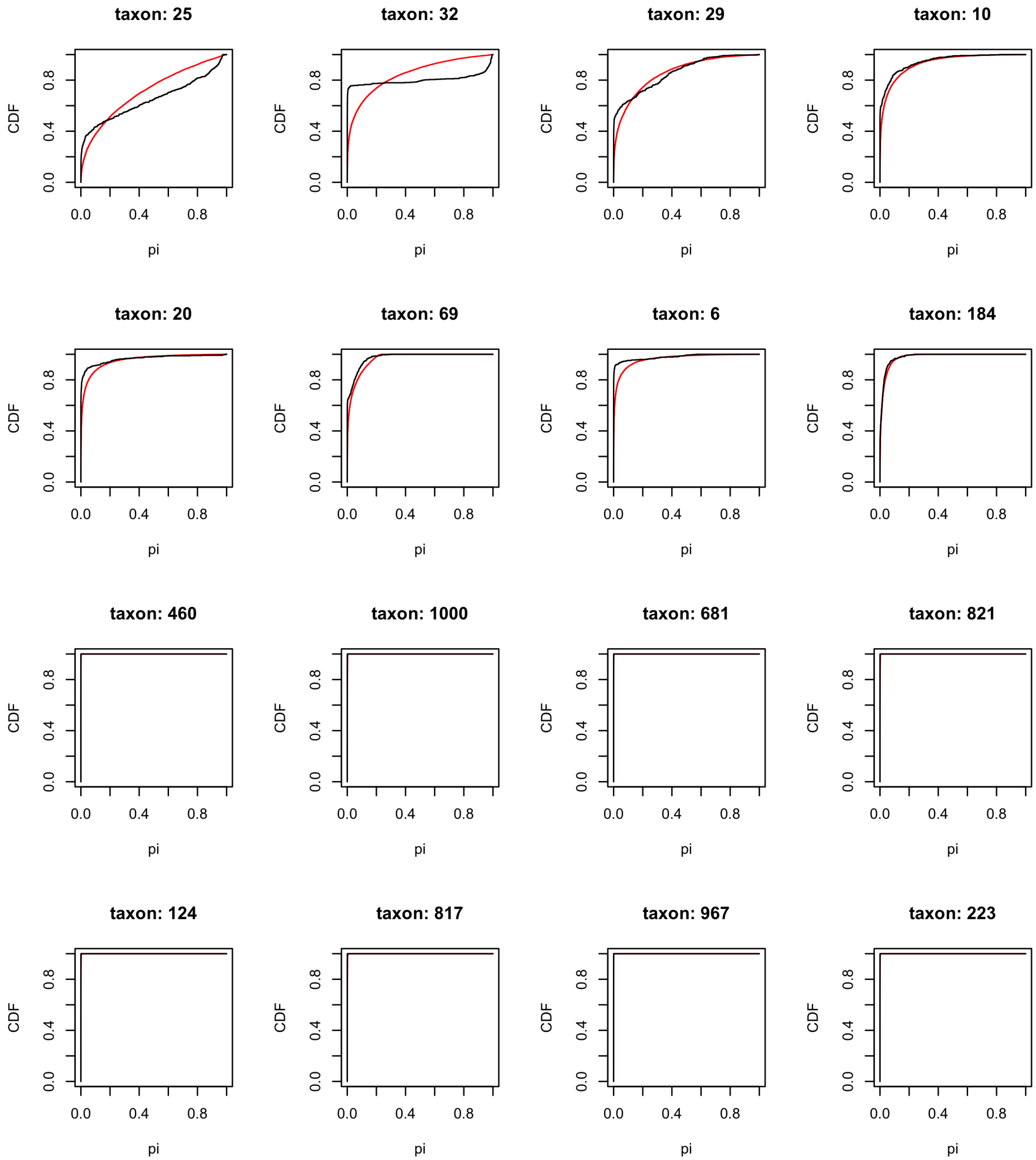

Figure S5: Comparison of the predicted (red) and empirical (black) estimates of the CDF of relative abundance for the top 8 and moderately abundant 8 taxa in MOMS-PI dataset.

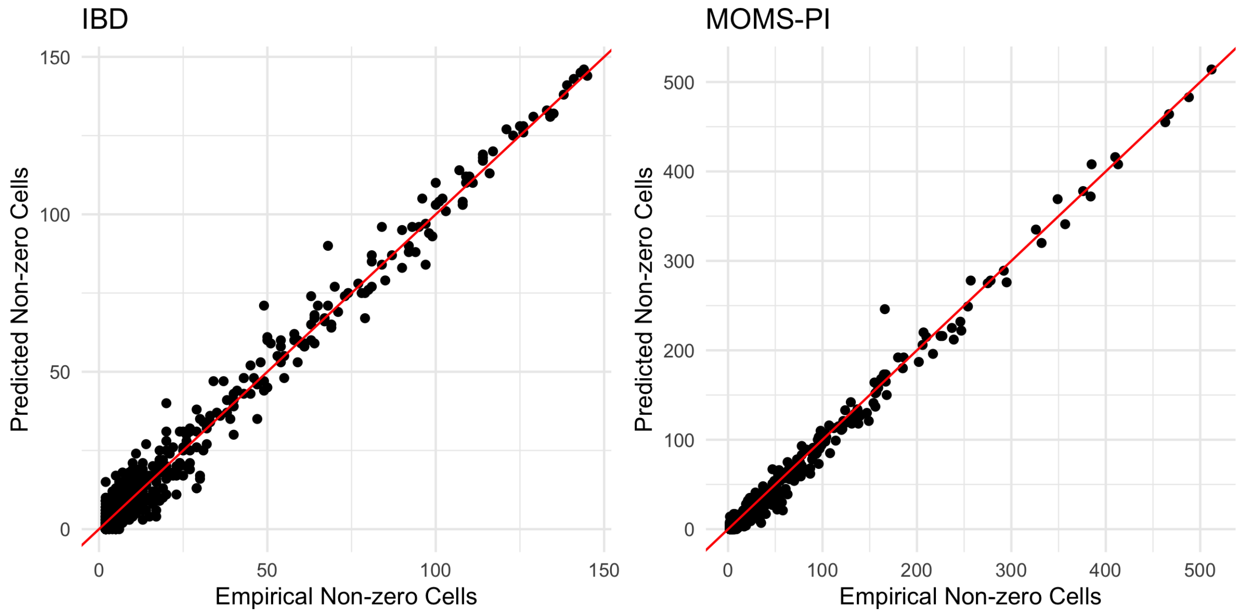

Figure S6: Comparison of the empirical Z_j and predicted $\tilde{Z}_j$ number of non-zero cells in IBD and MOMS-PI datasets. The red lines represent the diagonal reference lines.
